# Audit of Retinal Screening Protocol Adherence Among Patients Receiving Hydroxychloroquine at the Department of Rheumatology, Gulab Devi Teaching Hospital, Pakistan

**DOI:** 10.7759/cureus.115611

**Published:** 2026-09-01

**Authors:** Muhammad Mansoor Ali, Noor Ehsan, Ali Abbas Zahid, Masem Afzal, Usman Amjad, Muhammad Zubair

**Affiliations:** 1 Internal Medicine, Gulab Devi Teaching Hospital/Al-Aleem Medical College, Lahore, PAK; 2 Acute Medicine, Wrexham Maelor Hospital, Wrexham, GBR; 3 Rheumatology, Gulab Devi Teaching Hospital/Al-Aleem Medical College, Lahore, PAK

**Keywords:** clinical audit, clinical rheumatology, hydroxychloroquine, hydroxychloroquine retinopathy, internal medicine and rheumatology, ophthalmologic evaluation, quality improvement project, retinal hydroxychloroquine toxicity, retinal screening, screening guidelines

## Abstract

Introduction: Hydroxychloroquine (HCQ) is a widely used drug for various rheumatological conditions. Long-term use may be associated with retinal toxicity, which is often asymptomatic in the early stages and may lead to irreversible vision loss. Regular retinal screening is recommended in patients on long-term HCQ therapy. This audit assessed compliance with HCQ retinal screening protocols and evaluated changes in adherence following targeted interventions.

Materials and methods: A two-cycle clinical audit was conducted in the Department of Rheumatology, Gulab Devi Teaching Hospital, Lahore, Pakistan. The first cycle (August-October 2025) included 50 patients receiving HCQ therapy. Data were collected using a structured proforma through review of medical records and patient interviews. Three indicators, based on the 2016 American Academy of Ophthalmology (AAO) recommendations, were assessed: baseline retinal screening within one year of HCQ initiation, annual screening in patients on HCQ for ≥5 years, and early screening in high-risk patients (<5 years of therapy). High-risk patients were defined as those with one or more recognized risk factors for HCQ retinopathy, including an HCQ dose >5 mg/kg/day based on actual body weight, renal impairment, pre-existing macular disease, or concurrent tamoxifen use, and receiving HCQ for less than five years. Documentation of ophthalmology referral was also assessed. Interventions included patient counselling, educational leaflets, mandatory ophthalmology referral, structured documentation of high-risk status, and improved coordination with ophthalmology services. The second cycle (February-April 2026) included 50 new, non-overlapping patients assessed using the same methodology.

Results: In Phase 1, baseline retinal screening was documented in 12.0% (6/50), while 88.0% (44/50) had no record of screening. Annual screening in long-term users was 15.4% (4/26), and high-risk early screening was 9.1% (1/11). Ophthalmology referral was documented in 18.0% (9/50). Following interventions, baseline screening improved to 100% (50/50; p < 0.001). Annual screening improved to 76.0% (19/25; p < 0.001), and high-risk screening improved to 75.0% (9/12; p = 0.003). Ophthalmology referral improved to 100% (50/50; p < 0.001).

Conclusion: Baseline adherence to HCQ retinal screening was poor initially. Following structured interventions including counselling, referral pathways, and improved documentation, significant improvement was observed across all indicators. Some gaps remain in follow-up screening, particularly annual and high-risk monitoring, highlighting the need for continued reinforcement of guideline-based practice.

## Introduction

Hydroxychloroquine (HCQ) is a widely used disease-modifying antirheumatic drug (DMARD) for autoimmune conditions such as systemic lupus erythematosus (SLE), rheumatoid arthritis (RA), and other autoimmune diseases, offering proven efficacy in reducing disease activity and improving long-term outcomes [1]. However, long-term HCQ use carries the risk of retinal toxicity, classically known as bull’s-eye maculopathy [2]. This toxicity is thought to result from the accumulation of the drug within the lysosomes of the retinal pigment epithelial cells [3]. The chances of developing retinal toxicity become higher with longer duration of drug use and at higher drug doses, above 5 mg/kg actual body weight per day [4]. This toxicity is insidious and typically asymptomatic in early stages [5]. It can progress and lead to irreversible vision loss, making retinal screening necessary [6].

The American Academy of Ophthalmology (AAO) revised its recommendations in 2016, emphasizing the need for baseline screening within the first year of HCQ initiation and annual screenings for patients on the drug for five or more years, or earlier in high-risk cases (patients with renal impairment, on a high daily dose, or pre-existing retinal disease) [7]. The Royal College of Ophthalmologists (RCOphth) recommends retinal screening after five years of HCQ use, with earlier screening at the end of the first year of therapy for high-risk patients [8]. The RCOphth also advocates tailored monitoring protocols using advanced imaging modalities, such as spectral-domain optical coherence tomography (SD-OCT) and widefield fundus autofluorescence (FAF), to facilitate early detection of retinal toxicity [8]. 

The estimated prevalence of HCQ-related retinal toxicity is approximately 7.5% after more than five years of therapy and increases to around 20% after more than 20 years of treatment [9]. Despite clear recommendations, adherence to retinal screening remains suboptimal [10,11]. In Pakistan, data regarding adherence to HCQ retinal screening recommendations remain limited. This two-step audit was conducted to determine the percentage of patients being screened for HCQ-related retinal toxicity according to the American Academy of Ophthalmology (AAO) guidelines in a tertiary care hospital in Lahore, Pakistan. This study aims to evaluate compliance with AAO screening guidelines and to introduce and implement measures to improve retinal screening adherence.

## Materials and methods

Study design and setting

A two-cycle clinical audit was conducted in the Department of Rheumatology, Gulab Devi Teaching Hospital, Lahore, Pakistan, to assess adherence to retinal screening recommendations among patients receiving HCQ. The audit aimed to evaluate current practice, identify deficiencies in screening and documentation, implement corrective measures, and assess changes in practice following implementation of these interventions through re-audit.

Audit standards

The audit was conducted in accordance with the recommendations of the American Academy of Ophthalmology (AAO) 2016 revision [7]. We assessed documentation of baseline retinal screening within one year of HCQ initiation, annual retinal screening in patients receiving HCQ for five years or longer, and early retinal screening in high-risk patients. Baseline screening was assessed among all patients included in each audit cycle. High-risk patients were defined as those with one or more recognized risk factors for HCQ retinopathy, including an HCQ dose >5 mg/kg/day based on actual body weight, renal impairment, pre-existing macular disease, or concurrent tamoxifen use, and receiving HCQ for less than five years. Documentation of ophthalmology referrals was also evaluated. The denominator for each screening indicator was based on the patients eligible for that specific screening recommendation. Baseline screening was assessed among all patients included in each audit cycle (n = 50), irrespective of treatment duration, as it was based on documented evidence of screening within one year of HCQ initiation. Annual screening was assessed only among patients receiving HCQ for five years or longer, as this group was eligible for annual follow-up screening under the AAO 2016 recommendations. High-risk early screening was assessed only among patients receiving HCQ for less than five years who had one or more predefined risk factors for HCQ retinopathy, as these patients were eligible for earlier screening. 

Study population

Adult patients attending the rheumatology outpatient department and admitted to the inpatient department who were actively receiving HCQ for rheumatological conditions, including SLE, RA, and other autoimmune diseases, were eligible for inclusion. Patients who had discontinued HCQ therapy or had incomplete records that prevented assessment of retinal screening status were excluded. The two audit cycles included separate, non-overlapping groups of patients receiving HCQ therapy.

Data collection

The first audit cycle was conducted between August 2025 and October 2025 and included 50 consecutive patients receiving HCQ therapy. Data were collected using a structured audit proforma developed by the audit team (Appendix A and Appendix B). Information was obtained through review of medical records and patient interviews where necessary. The proforma captured demographic and clinical characteristics, HCQ treatment details, retinal toxicity risk factors, retinal screening status, and documentation of ophthalmology referral.​​​​​

Intervention phase

Following completion of the first audit cycle, the findings were presented to the rheumatology team, and several measures were introduced to improve compliance, including retinal screening recommendations. Clinicians were reminded of current screening guidelines and the importance of timely ophthalmology referral. Patients were counselled regarding the risk of HCQ-related retinal toxicity and the need for regular retinal examinations. Patients referred to the Ophthalmology Department underwent retinal assessment using optical coherence tomography (OCT), visual field testing, and fundus examination (fundoscopy), as appropriate. Educational leaflets explaining the importance of retinal screening were distributed during clinic visits (Appendix C). A standardized written referral system to the ophthalmology department was introduced for all patients receiving HCQ therapy. In addition, high-risk status was clearly documented in patient records to facilitate identification of patients requiring earlier surveillance. Coordination between the rheumatology and ophthalmology departments was strengthened to improve the completion of recommended retinal screening.

Repeat audit

A second audit cycle was conducted between February 2026 and April 2026 following implementation of the interventions. Fifty new patients receiving HCQ therapy were assessed using the same audit proforma and methodology used during the initial audit cycle. Compliance with the predefined audit standards was compared between the two cycles to evaluate the effect of the interventions.

Data analysis

Data were entered into Microsoft Excel (Microsoft Corp., Redmond, WA, USA) and analyzed using the IBM SPSS Statistics for Windows, Version 26.0 (Released 2018; IBM Corp., Armonk, NY, USA). Categorical variables were presented as frequencies and percentages. Compliance rates for each audit indicator were compared between the initial audit cycle and the re-audit to assess changes following implementation of the interventions. Comparisons involving the full cohort were performed using the Chi-square test, while Fisher's exact test was used for the high-risk subgroup because of the small sample size. A p-value < 0.05 was considered statistically significant.

## Results

A total of 100 patients receiving HCQ were included across two audit cycles, with 50 patients in each phase. The two audit cycles comprised separate, non-overlapping patient groups. SLE was the most common indication for HCQ use in both cycles, followed by RA and other autoimmune conditions, with a female predominance in the study population (Table 1). In the first audit cycle, baseline retinal screening within one year of HCQ initiation was documented in 12.0% (6/50) of patients, while 88.0% (44/50) had no evidence of appropriate baseline screening (Table 2). Following the implementation of targeted interventions, including patient counselling regarding HCQ-related retinal toxicity, distribution of educational leaflets, mandatory written referral to the ophthalmology department for all HCQ users, and improved documentation of high-risk status in patient records, baseline screening improved to 100% (50/50; p < 0.001) in the second cycle.

**Table 1:** Baseline characteristics of patients on hydroxychloroquine (HCQ)

Variable	Phase 1 (Aug-Oct 2025); N = 50	Phase 2 (Feb-Apr 2026); N = 50
Systemic lupus erythematosus	28 (56.0%)	29 (58.0%)
Rheumatoid arthritis	14 (28.0%)	15 (30.0%)
Other conditions	8 (16.0%)	6 (12.0%)
Urban residence	25 (50.0%)	28 (56.0%)
Rural residence	25 (50.0%)	22 (44.0%)
Male	8 (16.0%)	11 (22.0%)
Female	42 (84.0%)	39 (78.0%)
HCQ >5 mg/kg/day	28 (56.0%)	27 (54.0%)
HCQ ≤5 mg/kg/day	22 (44.0%)	23 (46.0%)
HCQ duration <5 years	24 (48.0%)	25 (50.0%)
HCQ duration ≥5 years	26 (52.0%)	25 (50.0%)

**Table 2:** Compliance with hydroxychloroquine (HCQ) retinal screening recommendations* *Baseline retinal screening within one year of hydroxychloroquine initiation was assessed in all patients receiving HCQ in each audit cycle (n = 50 per phase), irrespective of treatment duration. This included both newly initiated and long-term users, based on documented evidence of screening within the first year of therapy.

Parameter	Phase 1, N (%)	Phase 2, N (%)	p-value	Absolute change (percentage points)
Baseline screening within one year of HCQ initiation	6 (12.0%)	50 (100%)	<0.001	↑ 88.0 percentage points
No baseline screening	44 (88.0%)	0 (0%)	<0.001	↓ 88.0 percentage points
Annual screening in patients on HCQ ≥5 years	4/26 (15.4%)	19/25 (76.0%)	<0.001	↑ 60.6 percentage points
No annual screening in patients on HCQ ≥5 years	22/26 (84.6%)	6/25 (24.0%)	<0.001	↓ 60.6 percentage points
High-risk early screening (<5 years + risk factors)	1/11 (9.1%)	9/12 (75.0%)	0.003	↑ 65.9 percentage points
No high-risk early screening	10/11 (90.9%)	3/12 (25.0%)	0.003	↓ 65.9 percentage points
Ophthalmology referral documented	9 (18.0%)	50 (100%)	<0.001	↑ 82.0 percentage points

Among patients receiving HCQ for five years or more, who constituted the eligible population for the annual screening indicator, annual retinal screening was performed in 15.4% (4/26) in Phase 1, while 84.6% (22/26) were not screened as per recommendations. In the re-audit phase, compliance improved to 76.0% (19/25; p < 0.001), while 24.0% (6/25; p < 0.001) remained unscreened despite eligibility for annual follow-up. In patients identified as high-risk for HCQ-induced retinopathy and requiring earlier screening (n = 11, Phase 1; n = 12, Phase 2), appropriate screening was documented in 9.1% (1/11) during the initial cycle, which improved to 75.0% (9/12; p = 0.003) following interventions.

Documentation practices also showed marked improvement. Documentation of ophthalmology referral, a process measure introduced as part of the intervention, increased from 18.0% (9/50) in Phase 1 to 100% (50/50; p < 0.001) in Phase 2. Overall, a substantial improvement in adherence to HCQ retinal screening guidelines was observed across the measured domains following implementation of the interventions, particularly in baseline screening and referral practices, with residual gaps persisting mainly in long-term and high-risk follow-up screening. As the two audit cycles comprised different patient groups, these findings reflect changes observed in departmental screening practices between the two audit periods rather than longitudinal changes in the same patients. The denominators for each screening indicator reflected the number of patients eligible for that specific recommendation. Baseline screening was assessed in all 50 patients in each cycle, whereas annual screening was assessed only among patients receiving HCQ for ≥5 years, and high-risk early screening among patients receiving HCQ for <5 years with predefined risk factors. The absolute change in compliance was calculated as the difference between the Phase 2 and Phase 1 percentages. These values are reported as percentage-point changes rather than relative percentage changes.

## Discussion

Our study identified a significant baseline deficiency in adherence to HCQ retinal screening guidelines across all domains, including baseline, long-term, and high-risk monitoring. Initial compliance was particularly poor for baseline screening, long-term users, and high-risk patients, reflecting inadequate implementation of established AAO recommendations in routine clinical practice [7]. Following targeted interventions, a marked improvement was observed across all measured parameters. Baseline screening improved to full compliance, while long-term and high-risk screening also showed substantial improvement. 

The poor baseline adherence observed in the first audit cycle indicates a gap between AAO guideline recommendations and routine clinical workflows, particularly in long-term monitoring where sustained follow-up is required [7]. Similar deficiencies in adherence have been reported in an audit conducted at a rheumatology clinic in the United Kingdom by Worth et al., who identified significant gaps in adherence to screening guidelines, with a substantial proportion of patients exceeding safe dosing limits and lacking documentation of appropriate retinal screening [12]. Similarly, Islam et al. reported that only 55.3% of patients underwent retinal examinations at the recommended frequency after the 2016 AAO guideline update, while just 46.4% received macular optical coherence tomography at the recommended interval and only 21.0% underwent appropriate visual field screening [10].

Despite the overall improvement observed during the second cycle of our study, incomplete adherence remained in the long-term and high-risk groups, indicating persistent gaps in follow-up practice. Previous studies have revealed multiple other factors that may lead to poor retinal screening adherence. A 2023 population-based study by Kim et al. in South Korea, involving over 12,000 patients, identified treatment in primary care settings, older age, and lack of patient education as important factors associated with poor screening adherence [13]. Only 20.8% of the patient population had baseline retinal examinations within the recommended first year, and merely 17.6% underwent appropriate monitoring after five years of HCQ therapy [13]. A 2024 data analysis presented by Shahid et al. at the American College of Rheumatology Convergence found that only approximately 30% of patients with long-term HCQ use met AAO screening guidelines, with significant disparities observed in patients from underserved populations [14]. 

The results of our study are consistent with those of the study conducted by Khan et al. in a tertiary care hospital in Lahore, Pakistan [15]. Khan et al. reported that while baseline retinal screening was recommended for most patients, only 66% completed screening within the first year of HCQ initiation [15]. Adherence to five-year screening was poor, with only 22% of eligible patients undergoing SD-OCT, and advanced imaging modalities such as SD-OCT and FAF were rarely used [15]. Hence, our study builds on the limited local data and highlights the need for better screening adherence.

Overall, this audit provides important local evidence on HCQ retinal screening practices in Pakistan and found that implementation of targeted quality improvement measures was associated with a marked improvement in adherence to recommended screening practices. Because several interventions were introduced together, the individual contribution of each measure could not be determined. Furthermore, the two audit cycles included different, non-overlapping patient groups, and there was no concurrent control group; therefore, the observed improvement cannot be attributed definitively to the interventions and may reflect changes in departmental practice between the two audit periods. While further work is needed to address persistent gaps in long-term screening, the improvements observed in the re-audit highlight the value of regular clinical audit as a practical tool for improving the quality and safety of patient care [16].

Limitations

This audit has some limitations that should be considered when interpreting the findings. As with most quality improvement audits, awareness of the audit process may have influenced clinical practice during the re-audit period. The two cycles included different, non-overlapping patient groups, and there was no concurrent control group; therefore, the findings reflect changes observed in departmental screening processes rather than changes in individual patients over time, and a definitive causal effect of the interventions cannot be established. Although the groups were broadly comparable in their recorded baseline characteristics, unmeasured differences between the cohorts cannot be excluded. The overall sample size was relatively small, particularly in the high-risk subgroup, which may limit the precision of estimates for this group. Several screening indicators were assessed separately, and statistical comparisons were made without adjustment for multiple testing. In addition, patients who had discontinued HCQ were not included, which may have influenced the composition of the audited population. Despite these limitations, this two-cycle design provides useful real-world evidence of improvement associated with practical changes to screening and referral processes.

## Conclusions

This audit identified poor baseline adherence to recommended HCQ retinal screening guidelines in the initial cycle, particularly in baseline screening, follow-up of long-term users, and early screening of high-risk patients. Documentation of ophthalmology referral was also suboptimal, indicating gaps in both clinical practice and record-keeping.

Following implementation of targeted interventions, including clinician education, patient counselling, provision of educational leaflets, mandatory ophthalmology referral, and improved documentation of risk status, a marked improvement was observed across all audited domains. Baseline screening reached complete compliance, and substantial improvement was also seen in annual and high-risk screening as well as documentation practices. Despite this improvement, a proportion of patients in the re-audit cycle remained non-compliant with annual and high-risk screening recommendations, indicating ongoing gaps in follow-up adherence. As the two audit cycles included different, non-overlapping patient groups and there was no concurrent control group, the observed improvement should be interpreted as an association with the intervention period rather than definitive evidence of causation. This highlights the need for continued reinforcement of guideline-based practice, strengthened rheumatology-ophthalmology coordination, and sustained patient education to ensure long-term safety in HCQ use and prevent avoidable retinal toxicity.

## Figures and Tables

**Figure 1:**
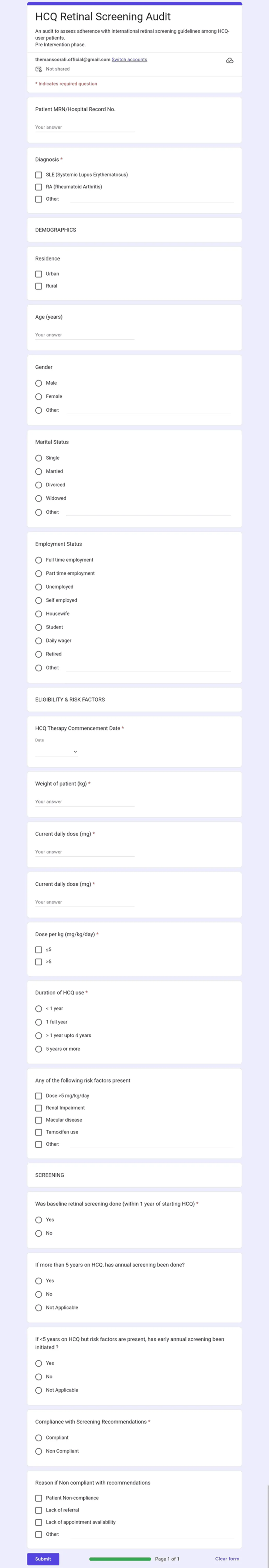
Pre-intervention data collection form

**Figure 2:**
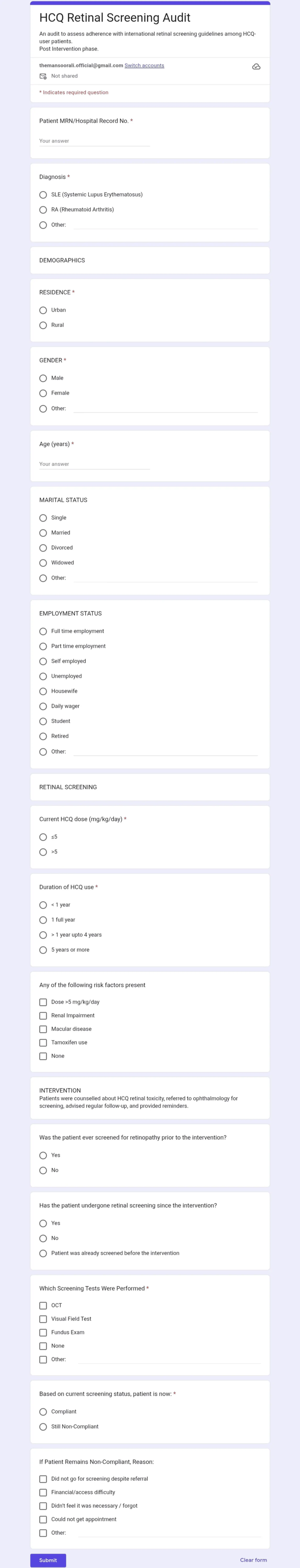
Post-intervention data collection form

**Figure 3:**
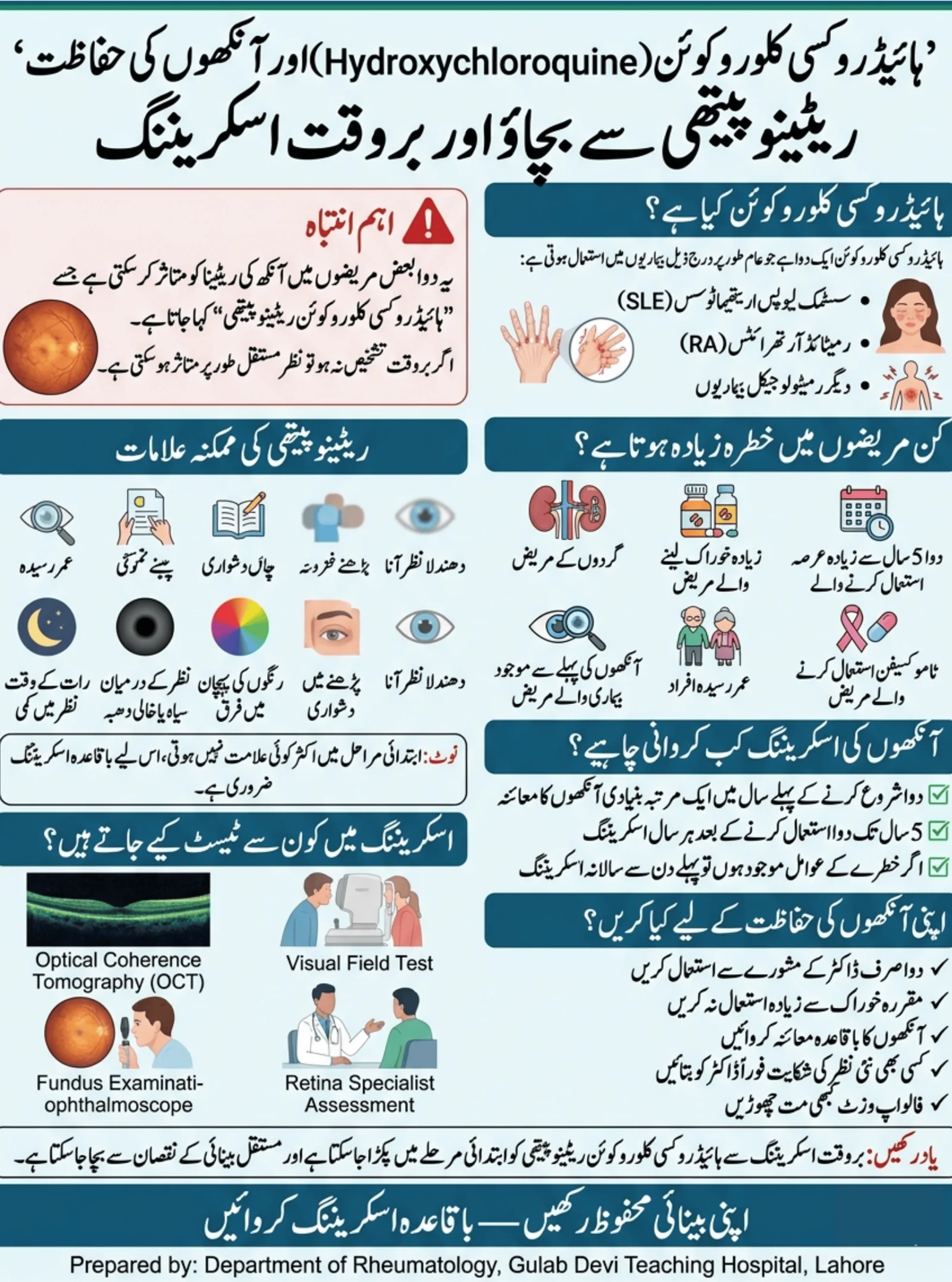
Patient education leaflet (in local language) Note: This leaflet was created by the research team itself; therefore, permission to use the image was obtained/authorized by the research team.

## Appendices

Appendix A

Appendix B

Appendix C 
